# Supplementary material for: Targeting Brain Tumors with Mesenchymal Stem Cells in the Experimental Model of the Orthotopic Glioblastoma in Rats
Source: Biomedicines. 2021 Nov 1;9(11):1592. doi: 10.3390/biomedicines9111592 (PMC8615766; doi:10.3390/biomedicines9111592)
Supplement: Supplementary file 1 [file biomedicines-09-01592-s001.zip › biomedicines-1426542-supplementary.pdf]

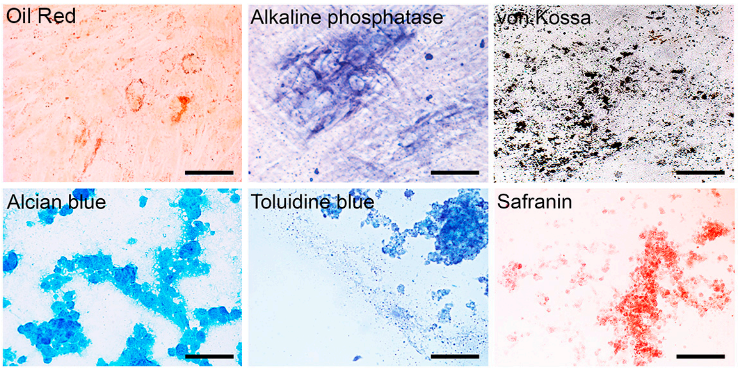

Figure S1. MSCs differentiation properties: Adipogenic differentiation. Stained with Oil Red; Osteogenic differentiation. Staining of alkaline phosphatase and von Kossa reaction; Chondrogenic differentiation. Staining with alcian and toluidine blue, and safranin, respectively. Scale bar, 100 μm.

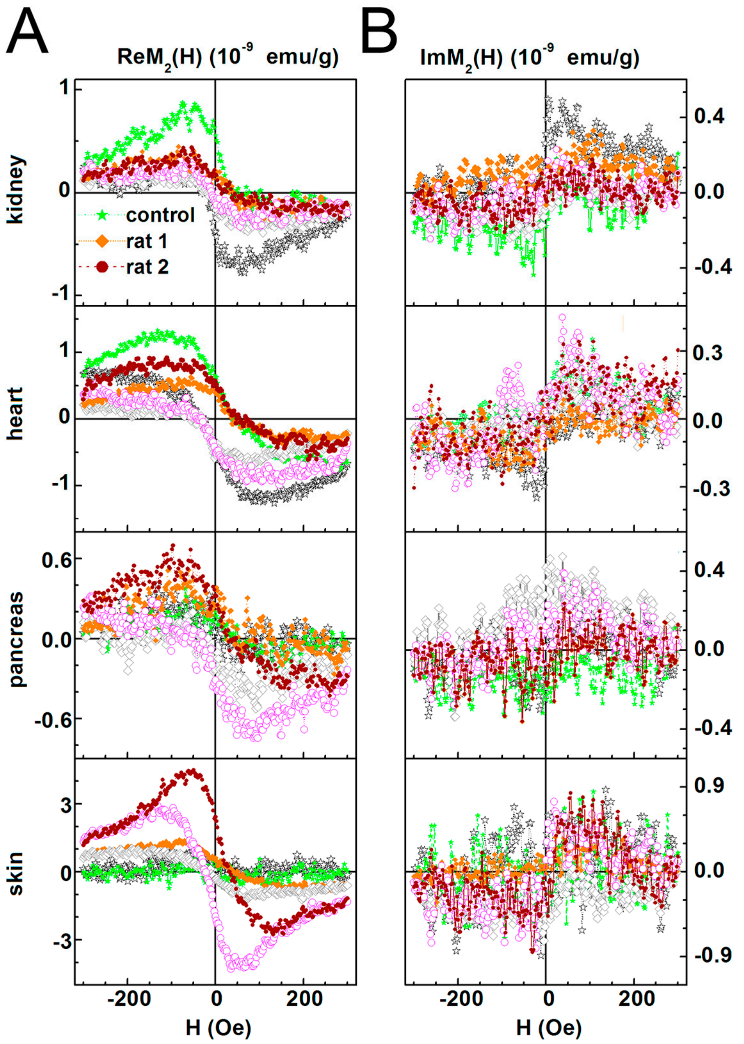

Figure S2. Real and imaginary parts of the nonlinear magnetic response as functions of the dc magnetic field direct (filled symbols) and reverse (open symbols) scans with $F_{sc} = 8$ Hz are presented for the kidney, heart, pancreas, and skin extracted from two rats at 24 h following injection of SPIONs-labeled MSCs and control rat without injection.

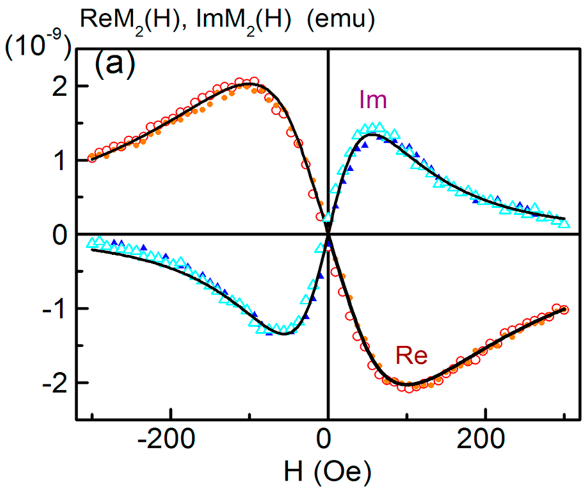

Figure S3. Real and imaginary parts of nonlinear magnetic response vs. dc magnetic field, direct (filled symbols) and reverse (open symbols) scans with F_{sc} = 8 Hz, for SPIONs-labeled MSCs suspensions in PBS after co-incubation of MSCs at the concentration of iron 150 μg/mL for 24 h. Every 32nd point is shown. Black curves are best fit. Obtained parameters are presented in Table 1.
